# Nationwide representative serosurvey of third-grade school children to evaluate the hepatitis B vaccination impact in Kyrgyzstan, 2022

**DOI:** 10.1186/s12879-025-10491-8

**Published:** 2025-01-22

**Authors:** Michael Brandl, Gulnara Zhumagulova, Gulbara Ishenapysova, Zuridin Nurmatov, Tatiana Enverovna Kuchuk, Nurzhan Zamirbekova, Gulsunai Sattarova, Saikal Temirbekova, Zhanara Bekenova, Martyna Gassowski, Liudmila Mosina, Antons Mozalevskis, Sandra Dudareva, Siddhartha Sankar Datta

**Affiliations:** 1 Department of Infectious Disease Epidemiology, Robert Koch Institute, Seestr. 10, 13353 Berlin, Germany; 2 Charité – Universitätsmedizin Berlin, corporate member of Freie Universität Berlin and Humboldt- Universität zu Berlin, Charitéplatz 1, 10117 Berlin, Germany; 3 Republican Center for Immunization, Ministry of Health of the Kyrgyz Republic, st. Frunze 535, Bishkek, 720033 Kyrgyz Republic; 4 National Institute of Public Health, Ministry of Health of the Kyrgyz Republic, st. Logvinenko 8, Bishkek, 720005 Kyrgyz Republic; 5 WHO Country Office for Kyrgyzstan, st. Orozbekova 52-54, Bishkek, 720040 Kyrgyz Republic; 6 WHO Regional Office for Europe, Marmorvej 51, Copenhagen, 2100 Denmark; 7 WHO Headquarters, Avenue Appia 20, Geneva, 1211 Switzerland

**Keywords:** Hepatitis B, Kyrgyzstan, Seroepidemiologic studies, Prevalence, Vaccination

## Abstract

**Background:**

Kyrgyzstan introduced universal hepatitis B childhood vaccination in 1999 to reduce the burden of hepatitis B. In 2016, aligned with the goal of controlling hepatitis B in the WHO European Region, a regional target of 0.5% was set for seroprevalence of hepatitis B surface antigen (HBsAg) among targeted birth cohorts. We conducted a representative nationwide serosurvey to assess the HBsAg prevalence among third-grade school children in Kyrgyzstan in 2022.

**Methods:**

We sampled numbers of children proportional to the population size and stratified the sample by region and urbanization level (urban/rural). We applied multistage cluster sampling with school classes as clusters. Identified participants in the survey were tested for HBsAg, using Enzyme-linked Immunosorbent Assay (ELISA), and positive samples confirmed with neutralization tests. Data on vaccination coverage for hepatitis B birth dose (HepB BD), including timing, and three doses of hepatitis B vaccine (HepB3) were collected from medical vaccination records. We calculated crude and weighted proportions for HBsAg seroprevalence and HepB BD and HepB3 coverage.

**Results:**

From the target sample size of 3,352 children, a total of 3,183 children (95%) participated in the survey. The majority of children were 9 or 10 years old (2,964; 93%) with almost equal numbers of girls and boys (1,606; 50% boys) and rural and urban participants (1,624; 51% urban). Five participants tested positive for HBsAg in confirmatory tests. The weighted HBsAg seroprevalence was 0.12% (95% CI 0.04–0.35%). Weighted coverage for HepB BD was 88% (95% CI 86–90%) and for HepB3 90% (95% CI 86–93%). Results from crude and weighted analysis did not differ statistically.

**Conclusions:**

Our study demonstrates the impact of a successfully implemented hepatitis B vaccination programme in Kyrgyzstan. High hepatitis B vaccination coverage has resulted in very low HBsAg seroprevalence among vaccinated birth cohorts, paving the way towards the achievement of regional hepatitis B control targets. Maintaining high vaccination uptake plus additional measures like screening of pregnant women and treatment of those infected will be key to achieve elimination of vertical transmission of hepatitis B in Kyrgyzstan.

**Supplementary Information:**

The online version contains supplementary material available at 10.1186/s12879-025-10491-8.

## Background

Hepatitis B is an infectious viral disease that may cause long-term damages to the liver like cirrhosis and hepatocellular carcinoma (HCC) [1]. Infections with hepatitis B virus (HBV) among new-borns and children often go asymptomatic but have the highest risk of causing chronic infections [2]. In 2016, the World Health Organization (WHO) called for ending viral hepatitis epidemics by the year 2030 in the Global Health Sector Strategy (GHSS) [3].

In the Global hepatitis report 2024, WHO estimated 10.6 million total hepatitis B infections in the European Region and identified Kyrgyzstan as a focus country for the viral hepatitis response [4]. The disease burden is particularly high in Central Asia and the death rate for liver complications due to hepatitis B was estimated to be seven times higher than in Western Europe in 2017 [5]. The first ever European action plan on viral hepatitis introduced targets for the control of hepatitis B through immunization in 2016, including the impact target of maximum 0.5% hepatitis B surface antigen (HBsAg) prevalence in vaccinated cohorts [6]. The action plans for ending AIDS and the epidemics of viral hepatitis and sexually transmitted infections 2022–2030 reinforced this intermediate target to be achieved by 2025 [7]. Representative serosurveys are the gold standard to assess prevalence and the results are used by the European Technical Advisory Group of Experts on Immunization (ETAGE) to validate countries for reaching hepatitis B control [8, 9]. Several countries in the WHO European Region already demonstrated the impact of vaccination with representative serosurveys, e.g. the Republic of Moldova in 2020 [10].

Kyrgyzstan introduced universal hepatitis B childhood vaccination in 1999 to prevent mother-to-child transmission (PMTCT) and early childhood transmission of hepatitis B [11]. The current vaccination schedule includes monovalent hepatitis B birth dose (HepB BD) administered within 24 h after birth and three doses (HepB3) with pentavalent vaccine (Diphtheria, Tetanus, Pertussis, Hepatitis B and Haemophilus ifluenzae type b) at 2, 3.5, and 5 months of age. Childhood vaccinations are provided by primary healthcare facilities to all children free of charge and since 2002, the vaccination coverage for both HepB BD and HepB3 among 1-year-olds exceeded 90% according to WHO/UNICEF estimates with only a small dip for HepB BD in 2011 [12]. During the COVID-19 pandemic, HepB BD coverage remained consistently high, but HepB3 coverage dropped under the 90% target in 2020 and 2021 with 86% and 89% coverage, respectively [12]. Further PMTCT measures like universal screening of pregnant women for hepatitis B have not yet been implemented in Kyrgyzstan and only when paid out of their own pocket, HBsAg testing during pregnancy is performed.

Reliable hepatitis B prevalence estimates from Kyrgyzstan are scarce and in 2018, the overall HBsAg prevalence in the Kyrgyz population was estimated at 2.2% [13]. A study published in 1992 before the introduction of the universal hepatitis B vaccination programme, with data from 979 participants from Osh region found HBsAg seropositivity of 10% overall and 15% among the under 1-year old children [14]. From 1998 to 2021, the incidence of acute HBV infections has drastically reduced from 28 to 2 per 100,000 population, with children under 18 years of age accounting for less than 1% of infections [15].

In order to generate evidence on the impact of hepatitis B vaccination in reducing new cases among vaccinated cohorts, the Ministry of Health of the Kyrgyz Republic (MoH), with the support from the WHO Regional Office for Europe and the Robert Koch Institute, Germany, performed the first nationwide hepatitis B serosurvey in Kyrgyzstan in 2022.

## Methods

### Study design

We conducted a cross-sectional serosurvey among children cohorts targeted by universal hepatitis B vaccination in Kyrgyzstan, following the WHO guidance on best practices for conducting a serosurvey to document the impact of hepatitis B immunization [8]. We applied stratified sampling by region and urbanization level and selected children in two stages. We used schools as sampling units and sampled children from school classes with a cluster size of 30 children.

### Study population

The target population of the study was 9–10-year-old children in Kyrgyzstan. As outlined in the WHO guidance, surveyed children should be between 5 and 15 years of age and born after the hepatitis B childhood vaccination had reached high levels of coverage for more than five years in a row [8]. We chose to sample children from one school grade corresponding to two age cohorts who would be 9 and 10 years old at the time of the study. In 2022, the average population size of the two age cohorts of children born in 2011 and 2012 was 147,283 children [16]. The sampled population were children enrolled in the third school grade of public schools in Kyrgyzstan in 2022. All children in selected classes were eligible for participation, given that an informed consent form signed by the parent or legal guardian was presented at the time of the study examination. There were no formal exclusion criteria.

### Sample size

We used the following formula to calculate the sample size (n) for a one-sided test with the significance level α and power 1-β:

$$\:n={\left(\frac{{z}_{1-\alpha\:}\sqrt{{p}_{0}\left(1-{p}_{0}\right)}\:+{\:z}_{1-\beta\:}\sqrt{{p}_{1}\left(1-{p}_{1}\right)}}{\delta\:}\right)}^{2}$$

$\:{p}_{0}$ = expected prevalence, $\:{p}_{1}$ = alternative prevalence (upper bound), $\:{z}_{1-\alpha\:}$ = quantile of the standard normal distribution, δ = difference between expected and alternative prevalence ($\:{p}_{1}$ - $\:{p}_{0}$), also called effect size. The α-level was set at 0.05 and the power (1-β) at 80%.

We set the design effect to 2 and considered it in a subsequent step of the sample size calculation.

Based on indications from available notification data and the three-dose vaccination coverage during the years 2010–2019, the prevalence in vaccinated cohorts was estimated at 0.30% for the purpose of sample size calculation [12, 17]. To ensure a feasible overall sample size, the upper precision bound for the national level was set at 0.69%. This resulted in a target sample size of 3,352 children.

### Study location and stratification

Kyrgyzstan is administratively divided into nine regions (seven *oblasts* plus two independent cities: Bishkek and Osh City), which are further divided into 40 *raions* (districts) plus 23 cities, both referred to as districts in this article [18]. We divided the total sample into three study areas with equally large sample sizes to estimate HBsAg seroprevalence in each area with an acceptable level of precision (Table 1).

**Table 1:** Sample size calculations, Kyrgyzstan, 2022

	Expected prevalence	Upper precision bound	Sample size
**Per area** (Bishkek, North, South)	0.30%	1.04%	1,118
**Total** (3 areas)	0.30%	0.69%	3,352

Values used for the sample size calculations: α = 0.05; Power (1-β) = 80%; Design effect = 2

We used the following division of the country on the regional level:

- Area (1) Bishkek (Bishkek City).
- Area (2) North (regions Chuy, Issyk-Kul, Naryn, and Talas),
- Area (3) South (regions Batken, Jalal-Abad, Osh City, and Osh Region).

In order to ensure that the selected sample proportionally reflected the distribution of the target population, we stratified the schools by both region and degree of urbanization. The classification of schools as either rural or urban was done according to official data of the National Statistical Committee of the Kyrgyz Republic [16]. All schools in Bishkek and Osh City were classified as urban. Stratification of the sample resulted in a total of 16 strata: one in Bishkek, eight in area North and seven in area South.

### Sampling

The number of clusters sampled from each stratum was proportional to the population size of third-grade children. This gave a total of 116 clusters. As a first step, schools were selected using probability-proportional-to-size sampling. As a second step, classes from the selected schools were selected using simple random sampling. We assumed a response rate of 80%, based on national expert opinion in Kyrgyzstan. This resulted in 38 children per cluster to be invited, or a total N of 3,964 children. In cases where there were between 30 and 38 children in a class and there were sufficient test kits, the whole class was invited to participate. However, most clusters only had material to test 30 children. We calculated participation rates per stratum and area, which therefore reflect both the response and the limited availability of test kits.

### Recruitment of participants

Children were recruited through schools, with parents or legal guardians, directors and school teachers informed in advance. Parents received a letter of invitation, information about the study, and an informed consent form. All parents returned the consent form in a closed envelope, to protect the confidentiality of the decision of the parents, at the latest the day before the blood sample collection. The consent forms for all children were collected by class teachers, who kept records of the number of forms returned.

### Laboratory testing procedure and algorithm

All samples were tested for HBsAg using Enzyme-linked Immunosorbent Assay (ELISA). HBsAg-positive samples were retested to confirm presence of HBsAg by neutralization tests. All testing was performed in the national reference laboratory, the Laboratory of the National Institute of Public Health of the MoH. Testing was carried out in strict accordance with the instructions of the manufacturer of the Murex HBsAg Version 3 and Murex HBsAg Confirmatory Version 3 (DiaSorin, UK) test systems and standard operating procedures of ELISA testing. Murex HBsAg Version 3 assay sensitivity is 100% (95% CI 98.2–100%) and specificity is 99.0% (95% CI 97.2–99.8%) [19]. Prior to this study, we established a sensitivity of 0.05 IU/ml using the third international standard (HBV genotype B4, HBsAg subtypes ayw1/adw2). For the second international standard HBsAg genotype A HBsAg subtypes adw, the analytical sensitivity was 0.25 IU/ml.

### Data collection

We developed a questionnaire for data collection based on the WHO guidance on conducting hepatitis B serosurveys [8]. From all participating children, demographic data and vaccination information were collected by school nurses or nurses working at the local primary healthcare facility affiliated with the respective school. Demographic data were extracted from patient files and included age, sex, urbanization level, and region. Vaccination information were collected from medical vaccination records and included coverage of birth dose and timing, as well as coverage of first, second, and third doses. In case no vaccination information was available at schools, study personnel checked with the affiliated healthcare facility. If also not available there, vaccination information was marked as not available.

Venous blood samples from all participants were collected in 4 ml vacuum tubes at schools by nurses and sent within 24 h to the National Institute of Public Health of the MoH (samples from regions Bishkek and North) or to Osh Virology Laboratory (samples from region South). Samples at Osh Virology Laboratory were subsequently sent to the National Institute of Public Health, where all samples were stored at -80 °C until HBsAg testing.

In order to assess the extent of selection bias, demographic and vaccination information was also collected from “refusals”, children who were selected for the survey but who did not participate in the survey and from “classmates”, non-participating children who went to the same school classes as participants and refusals, but who were not selected for the survey.

### Data analysis

We calculated participation rates and proportions of the target sample size reached for the entire sample, each study area, and each stratum. Using a map of Kyrgyzstan, we displayed numbers of participants per study area and region, plus the number of clusters, which were selected per district.

We described participants and refusals by age, sex, urbanization level, and region. We compared the two groups with Wilcoxon rank sum and Pearson’s Chi-squared test and calculated *p*-values. We reported vaccination coverage for all four hepatitis B vaccine doses for participants, refusals, and classmates. We compared participants with refusals and participants with non-selected classmates pairwise using Pearson’s Chi-squared test.

We estimated the HBsAg seroprevalence, HepB BD coverage, and HepB3 coverage using the average population size of the age cohorts of children born in 2011 and 2012. We applied post-stratification weighting to account for differences in participation rates and population sizes and considered the study design in the analysis to yield crude and weighted proportions. We calculated the design effect of the analysis of HBsAg seroprevalence, which indicates the influence of the study design on the outcome. Additionally, we calculated weighted results stratified by sex, urbanization level, and study area.

## Results

### Study population

**Fig. 1:**
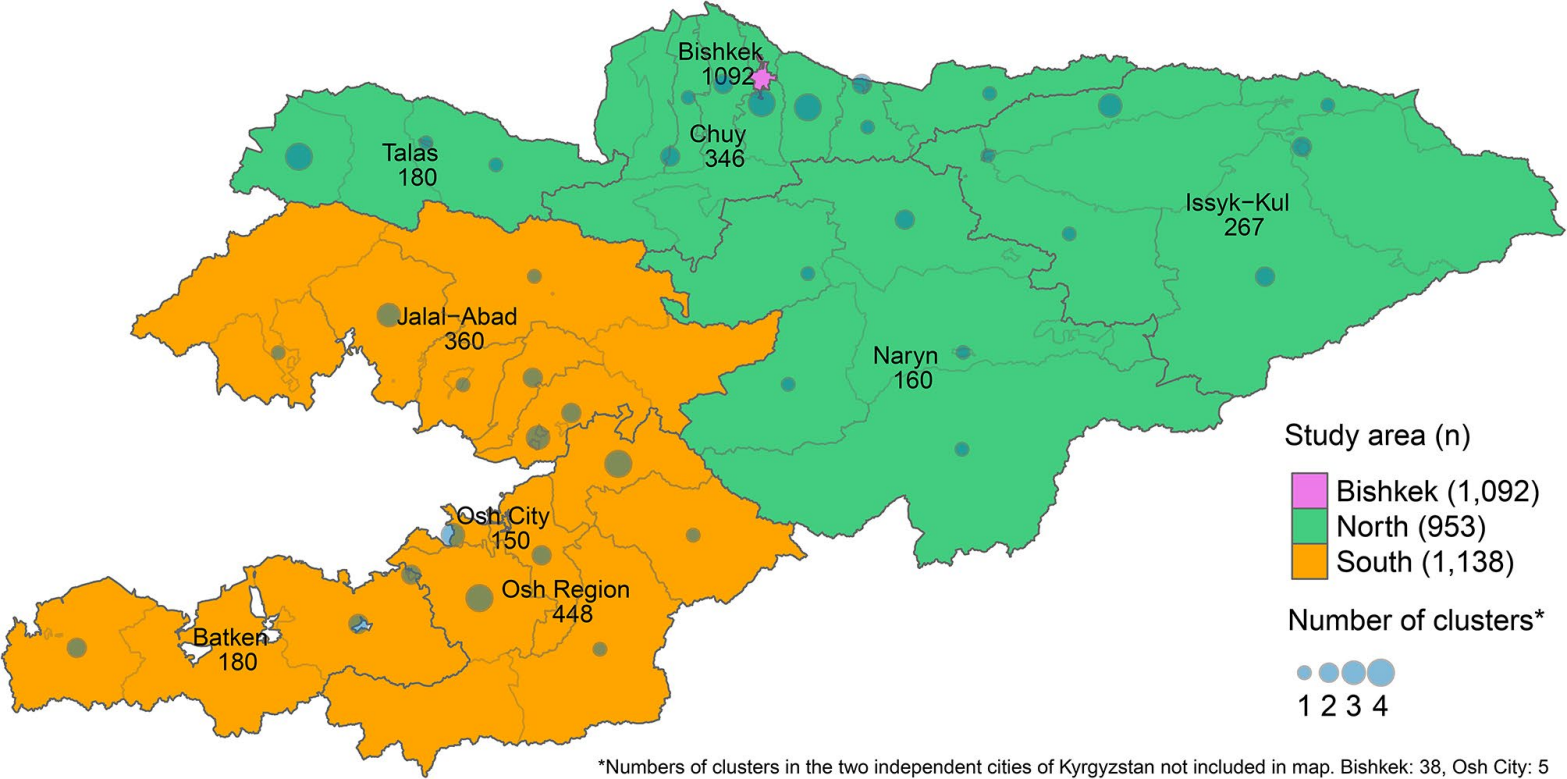
Numbers of participants (n) per study area and region and numbers of clusters per district, Kyrgyzstan, 2022

A total of 3,183 children participated in the survey, which relates to 95% reached of the target sample size of 3,352. Numbers of participants per study area and region, as well as numbers of clusters drawn per district are displayed in Fig. 1. In area Bishkek, participation was highest with 95% and in area South it was lowest with 72%. The highest proportion of the target sample size was reached in area South with 102% and the lowest in area North with 85%. Details on participation rates and proportions of sample size reached across study areas and strata are presented in Table A1 in the Additional file 1.

### Descriptive results

The majority of children were 9 or 10 years old (93%; 2,964/3,183). There were slightly more boys (50%; 1,606/3,183) than girls (50%; 1,577/3,183) in the final sample (Table 2). Participants and refusals were similar in regards to age (*p* = 0.2) and sex, but refusals were more often from rural schools and from regions in study area South.

**Table 2:** Characteristics of study participants and refusals, Kyrgyzstan, 2022

Variable	Value	Participants (*n* = 3,183)		Refusals (*n* = 781)		*p*-value^1^
		*n*	%	*n*	%	
Sex	Female	1,577	50%	369	47%	0.3
	Male	1,606	50%	411	53%	
Urbanization level	Urban	1,624	51%	283	36%	< 0.001
	Rural	1,559	49%	498	64%	
Region	Bishkek	1,092	34%	61	7.8%	< 0.001
	Chuy	346	11%	124	16%	
	Issyk-Kul	267	8.4%	57	7.3%	
	Naryn	160	5.0%	8	1.0%	
	Talas	180	5.7%	87	11%	
	Batken	180	5.7%	100	13%	
	Jalal-Abad	360	11%	131	17%	
	Osh Region	448	14%	160	20%	
	Osh City	150	4.7%	53	6.8%	

^1^ Comparison of refusals with participants using Pearson’s Chi-squared test

We found five cases who were tested positive for HBsAg and confirmed. Cases were predominantly male (80%; 4/5) and went to urban schools (60%; 3/5). The cases were found in the regions Bishkek (60%; 3/5), Naryn (20%; 1/5), and Osh Region (20%; 1/5).

Participants had higher coverage for all four doses of hepatitis B vaccine compared to refusals (Table 3). Among participants with information on vaccinations, 365 (12%; 365/3,143) had not received HepB BD and 308 (9.8%; 308/3,146) had not received HepB3. The vaccination coverage of classmates was similarly high for all four doses compared to participants. All vaccinated children had received monovalent hepatitis B vaccines as birth dose and pentavalent vaccines for subsequent doses.

**Table 3:** Vaccination coverage of study participants, refusals, and classmates, Kyrgyzstan, 2022

		Participants (*n* = 3,183)		Refusals (*n* = 781)		*p*-value^1^	Classmates (*n* = 1,426)		*p*-value^2^
Hepatitis B birth dose	vaccinated	2,778	88%	639	83%	< 0.001	1,268	89%	0.4
	not vaccinated	365	12%	130	17%		153	11%	
Time of administration of birth dose	day of birth	1,428	52%	359	58%	< 0.001	517	44%	0.03
	one day after birth	1,025	38%	212	34%		427	37%	
	more than one day	269	9.9%	48	7.8%		220	19%	
	(implausible or missing date of administration or birth)	(461)		(162)			(262)		
Hepatitis B first dose	vaccinated	2,945	94%	682	89%	< 0.001	1,354	95%	0.06
	not vaccinated	202	6.4%	87	11%		71	5.0%	
Hepatitis B second dose	vaccinated	2,923	93%	681	89%	< 0.001	1,337	94%	0.2
	not vaccinated	223	7.1%	88	11%		87	6.1%	
Hepatitis B third dose	vaccinated	2,838	90%	662	86%	0.002	1,269	89%	0.3
	not vaccinated	308	9.8%	105	14%		153	11%	

^1^ Comparison of refusals with participants using Pearson’s Chi-squared test

^2^ Comparison of non-participating classmates with participants using Pearson’s Chi-squared test

Of the participants with HepB BD with data on time of administration, 2,453 (90%; 2,453/2,722) had received the vaccine within one day after birth and 269 (9.9%; 269/2,722) more than one day after birth. The proportion of late administration of HepB BD was lower among refusals (7.8%; 48/619) and higher among classmates (19%; 220/1,164). All five HBsAg-positive cases had received timely HepB BD (three on the same day and two on the next day) and three additional doses of hepatitis B vaccine. We did not find any HBsAg-positive cases among unvaccinated participants.

### Weighted analysis of hepatitis B seromarkers

Eleven blood samples could not be tested, in five cases because of too little blood volume and in six cases because of haemolysis of samples, resulting in a total of 3,172 samples analysed. We found an overall weighted HBsAg prevalence estimate of 0.12% (95% CI 0.04–0.35%) HBsAg, which did not differ statistically from the crude proportion (Table 4). The design effect was calculated at 1.1.

Weighted proportion for HepB BD was 88% (95% CI 86–90%) and for HepB3 90% (95% CI 86–93%) and did not differ from crude proportions.

**Table 4:** Analysis of HBsAg seroprevalence and hepatitis B vaccination coverage, Kyrgyzstan, 2022

	*n*	Crude proportion	95% CI	Weighted proportion	95% CI
HBsAg seroprevalence estimate	5	0.16%	0.07–0.38%	0.12%	0.04–0.35%
Hepatitis B birth dose coverage	2,778	88%	87–89%	88%	86–90%
Hepatitis B third dose coverage	2,838	90%	89–91%	90%	86–93%

CI = confidence interval, HBsAg = hepatitis B surface antigen

Prevalence estimates did not differ statistically between sex, urbanization level, and study areas but were numerically higher in boys (0.21%; 95% CI 0.07–0.68%) and in study area Bishkek (0.27%; 95% CI 0.06–1.21%). Vaccination coverage for HepB BD was highest in rural locations (90%; 95% CI 86–92%) and study area North (92%; 95% CI 89–94%) with similar results for HepB3 (Table 5).

**Table 5:** Stratified results of weighted analysis of hepatitis B surface antigen positive participants and hepatitis B vaccination coverage, Kyrgyzstan, 2022

Variable	Value	Weighted HBsAg-positive proportion	95% CI	Weighted HepB BD coverage	95% CI	Weighted HepB3 coverage	95% CI
Sex	Female	0.03%	0.01–0.23%	88%	85–91%	89%	84–92%
	Male	0.21%	0.07–0.68%	88%	85–90%	91%	87–94%
Urbanization level	Urban	0.12%	0.03–0.53%	85%	81–89%	89%	80–94%
	Rural	0.13%	0.03–0.53%	90%	86–92%	91%	87–94%
Study area	Bishkek	0.27%	0.06–1.21%	88%	84–91%	89%	83–93%
	North	0.10%	0.01–0.75%	92%	89–94%	93%	90–94%
	South	0.09%	0.01–0.69%	86%	81–89%	89%	81–94%
**Total**		**0.12%**	**0.04–0.35%**	**88%**	**86–90%**	**90%**	**86–93%**

CI = confidence interval, HBsAg = hepatitis B surface antigen, HepB BD = hepatitis B birth dose, HepB3 = hepatitis B third dose coverage

## Discussion

This serosurvey provided valuable data on the significant impact of vaccination in reducing the prevalence of chronic hepatitis B in Kyrgyzstan. The obtained evidence adds to the growing proof of benefits of immunization and will be valuable for the MoH and National Immunization Programme (NIP) to advocate for sustainable financing. It also supports efforts to increase demand for vaccination. Additionally, the data from this study could help to estimate the number of cases of chronic hepatitis B and hepatitis B-related HCC and liver cirrhosis averted by vaccination, along with associated savings in treatment costs.

Based on consistently reported high hepatitis B coverage prior to the COVID-19 pandemic and significant national efforts to implement catch-up vaccination and improve immunization coverage afterwards, as well as the low HBsAg seroprevalence found in this study, ETAGE validated that Kyrgyzstan has reached regional targets for control of hepatitis B through immunization in November 2023 [20]. High vaccination uptake with both HepB BD and HepB3 contributed to this success by prevention of infections acquired perinatally and during childhood. All children in our study were in age groups that had already benefitted from implemented universal hepatitis B vaccination, which had reached high coverage. As a next step, Kyrgyzstan plans to intensify efforts to increase coverage of hepatitis B and other childhood vaccines, which has declined since the COVID-19 pandemic, and sustain at high levels to ensure equitable access to all children. Additionally, a vaccination campaign for adults was launched in January 2023, offering vaccination to all citizens of Kyrgyzstan from 23 to 65 years of age, indicating also key populations like prisoners, people who inject drugs, men who have sex with men, transgender people, as well as healthcare workers and household or sexual contacts of people with chronic hepatitis B [21].

The timeliness of hepatitis B birth dose is a critical factor in maximizing the impact of hepatitis B vaccination. To maintain strong control of hepatitis B and make progress toward eliminating it as a public health problem – aligned with the Regional action plans for ending AIDS and the epidemics of viral hepatitis and sexually transmitted infections 2022–2030 – the NIP should continue efforts to improve the timely administration of hepatitis B birth dose and to closely monitor this indicator [7]. In addition, Kyrgyzstan should consider introducing universal screening for HBsAg of pregnant women. Antiviral prophylaxis should then be offered to HBsAg-positive women with high viral load, who are positive for hepatitis B e antigen (HBeAg), or when additional testing for HBV DNA or HBeAg is not available [22, 23].

We found only five cases of chronic HBV infection. Four of the five cases were male; however, our study was not powered to reveal statistical differences between sexes. Differences between sexes have also been shown in other serosurveys, e.g. in the Republic of Moldova, which was also not powered to asses this effect [10]. Still, boys may be at higher risk of infection and male sex and gender be associated with increased risk of chronic HBV infection [24]. Furthermore, female sex has been found to be associated with higher vaccine-induced immunity [25].

Cases did not differ in regards to urbanization level, region, or school and were all vaccinated within one day after birth. In rare cases, transmission of HBV can still occur despite timely and full vaccination of new-borns and infants [26, 27]. It was out of the scope of our study to assess the hepatitis B serostatus of mothers and close contacts of HBsAg-positive children. As Kyrgyzstan does not implement universal screening of pregnant women for hepatitis B, it is likely that infected mothers with potentially high viral load might not have received antiviral treatment during their pregnancy and thus the infection might have happened intrauterine before birth [28].

Our results are in line with similar studies conducted in other countries of the WHO European Region. In the Republic of Moldova, which introduced universal hepatitis B vaccination in 1989, the HBsAg prevalence was estimated at 0.21% (95% CI 0.08–0.53%) in 2020 in a representative serosurvey [10]. In 2021, a serosurvey in Georgia among 1,473 5–17 year olds found the very low HBsAg prevalence of only 0.03% [29]. In Uzbekistan, a nationwide serosurvey among first to third-grade school children estimated the seroprevalence at 0.20% (95% CI 0.09–0.38%) in 2022 [30]. In Tajikistan, the seroprevalence was estimated at 0.4% (95% CI 0.1–1.3%) among 1-6-year-old children in 2010, when HepB3 coverage was already over 80% [31]. By the end of 2023, a total of nine countries in the WHO European Region have been validated for achievement of hepatitis B control targets [9, 32].

We collected vaccination information not only from participants, but also from non-participants and classmates to assess possible selection bias. Refusals had up to five per cent lower coverage for the four doses of hepatitis B vaccine. This seems plausible, as parents who refuse to get their children vaccinated may be more cautious also to have them participate in a study that includes a venous blood draw. Not invited classmates on the other hand did not differ in vaccination coverage from the participating children. This shows, that the children within selected classes were chosen randomly and selection of children was not influenced by vaccination information.

### Limitations

Our study has a few limitations. With our cross-sectional study design, we cannot prove a causal link between the introduction of the hepatitis B vaccination programme and the drastically reduced HBsAg prevalence. Furthermore, we cannot make statements about the HBsAg seroprevalence of children, who did not participate in the survey. Their HBsAg seropositivity could potentially be higher as their vaccination coverage was lower. However, we do not expect that this has introduced major bias in the study results, as all five positive cases were found among vaccinated children.

Our study slightly missed the target sample size and the participation rate was limited predominantly by availability of vacuum tubes. With 95% of the target sample size reached, we still consider our study adequately representative for the age cohorts of children born in 2011/2012. The sampling population included only children from public schools and no other school types like private schools.

It was out of the scope of our study to determine hepatitis B serostatus of participants’ mothers or additional hepatitis B seromarkers like anti-HBc which might have unveiled exposure to HBV. The hepatitis B vaccination data collected from study participants may underestimate the actual coverage, as these children were vaccinated 9–10 years ago. Records may have been lost or omitted if health facilities issued new immunization cards to replace lost ones or if children relocated to different areas, leading to incomplete or missing vaccination information.

## Conclusions

Our study demonstrates the impact of a successfully implemented hepatitis B vaccination programme in reducing HBsAg prevalence in vaccinated cohorts in Kyrgyzstan. Other countries in the WHO European Region should be encouraged to conduct serosurveys and ascertain the prevalence of HBsAg, thereby documenting the impact of hepatitis B vaccination and validating achievement of regional hepatitis B control targets. Maintaining high coverage with hepatitis B vaccine in every community in Kyrgyzstan will be essential to maintain hepatitis B control. Additional measures like screening of pregnant women and treatment of those infected will be key to prevent more cases, reduce mortality, and reach the hepatitis B elimination goal.

## Electronic supplementary material

Below is the link to the electronic supplementary material.

Supplementary Material 1

## Data Availability

The datasets used and analysed during the current study are available from the corresponding author upon reasonable request.
