# Supplementary material for: Nationwide representative serosurvey of third-grade school children to evaluate the hepatitis B vaccination impact in Kyrgyzstan, 2022
Source: BMC Infect Dis. 2025 Jan 22;25:100. doi: 10.1186/s12879-025-10491-8 (PMC11752756; doi:10.1186/s12879-025-10491-8)
Supplement: Supplementary file 1 — Supplementary Material 1 [file 12879_2025_10491_MOESM1_ESM.docx]

***Additional file 1: Supplementary material***

Table A1: Number and proportions of sampled and participating children by area and strata, Kyrgyzstan, 2022

| Region | Target number of participants | Number of sampled participants | Number of participating children | Participation ratio | Percentage of target number reached |
| --- | --- | --- | --- | --- | --- |
| Kyrgyzstan total | 3,352 | 3,964 | 3,183 | 80% | 95% |
| Area: |  |  |  |  |  |
| Bishkek | 1,118 | 1,153 | 1,092 | 95% | 98% |
| North | 1,118 | 1,229 | 953 | 78% | 85% |
| South | 1,118 | 1,582 | 1,138 | 72% | 102% |
| Strata: |  |  |  |  |  |
| Bishkek-Urban | 1,118 | 1,153 | 1,092 | 95% | 98% |
| Chuy-Urban | 106 | 121 | 76 | 63% | 72% |
| Chuy-Rural | 400 | 349 | 270 | 77% | 68% |
| Issyk-Kul-Urban | 80 | 90 | 66 | 73% | 83% |
| Issyk-Kul-Rural | 205 | 234 | 201 | 86% | 98% |
| Naryn-Urban | 26 | 37 | 30 | 81% | 115% |
| Naryn-Rural | 144 | 131 | 130 | 99% | 90% |
| Talas-Urban | 29 | 30 | 30 | 100% | 103% |
| Talas-Rural | 131 | 237 | 150 | 63% | 115% |
| Batken-Urban | 50 | 78 | 60 | 77% | 120% |
| Batken-Rural | 118 | 202 | 120 | 59% | 102% |
| Jalal-Abad-Urban | 65 | 142 | 90 | 63% | 138% |
| Jalal-Abad-Rural | 265 | 349 | 270 | 77% | 102% |
| Osh Region-Urban | 58 | 53 | 30 | 57% | 52% |
| Osh Region-Rural | 420 | 555 | 418 | 75% | 100% |
| Osh City-Urban | 146 | 203 | 150 | 74% | 103% |
